# Accumulation of Catechin and Proanthocyanidins in Black Poplar Stems After Infection by *Plectosphaerella populi*: Hormonal Regulation, Biosynthesis and Antifungal Activity

**DOI:** 10.3389/fpls.2019.01441

**Published:** 2019-11-15

**Authors:** Chhana Ullah, Sybille B. Unsicker, Michael Reichelt, Jonathan Gershenzon, Almuth Hammerbacher

**Affiliations:** ^1^Department of Biochemistry, Max Planck Institute for Chemical Ecology, Jena, Germany; ^2^Department of Zoology and Entomology, Forestry and Agricultural Biotechnology Institute, University of Pretoria, Pretoria, South Africa

**Keywords:** chemical defense, cytokinin, flavonoids, phytohormones, *Populus*, salicylic acid, secondary metabolism

## Abstract

Flavan-3-ols including the monomeric catechin and the polymeric proanthocyanidins (PAs) are abundant phenolic metabolites in poplar (*Populus* spp.) previously described to protect leaves against pathogen infection. However, it is not known whether stems are also defended in this way. Here we investigated flavan-3-ol accumulation, activity, and the regulation of formation in black poplar (*P. nigra*) stems after infection by a newly described fungal stem pathogen, *Plectosphaerella populi*, which forms canker-like lesions in stems. We showed that flavan-3-ol contents increased in *P. populi*-infected black poplar stems over the course of infection compared to non-infected controls. Transcripts of leucoanthocyanidin reductase (*LAR*) and anthocyanidin reductase (*ANR*) genes involved in the last steps of flavan-3-ol biosynthesis were also upregulated upon fungal infection indicating *de novo* biosynthesis. Amending culture medium with catechin and PAs reduced the mycelial growth of *P. populi*, suggesting that these metabolites act as anti-pathogen defenses in poplar *in vivo*. Among the hormones, salicylic acid (SA) was higher in *P. populi*-infected tissues compared to the non-infected controls over the course of infection studied, while jasmonic acid (JA) and JA-isoleucine (JA-Ile) levels were higher than controls only at the early stages of infection. Interestingly, cytokinins (CKs) were also upregulated in *P. populi*-infected stems. Poplar saplings treated with CK showed decreased levels of flavan-3-ols and SA in stems suggesting a negative association between CK and flavan-3-ol accumulation. Taken together, the sustained upregulation of SA in correlation with catechin and PA accumulation suggests that this is the dominant hormone inducing the formation of antifungal flavan-3-ols during *P. populi* infection of poplar stems.

## Introduction

Poplars (*Populus*, Salicaceae) are fast-growing trees native to the Northern hemisphere and are of economic and ecological importance (Hansen, 1993; Updegraff et al., 2004; Truax et al., 2012). Widely cultivated poplar species are considered excellent model organisms for woody plant research due to their fast growth, clonal reproduction, availability of the complete genome of *P. trichocarpa* and established genetic, as well as biochemical tools (Tuskan et al., 2006; Jansson and Douglas, 2007). Under natural conditions, poplars are often challenged by a plethora of biotrophic and necrotrophic fungal pathogens (Phillips and Burdekin, 1982; Newcombe et al., 2001). One of the most destructive and best-studied poplar pathogens is *Melampsora* spp. (poplar rust), which reduces photosynthesis, and causes early defoliation resulting in decreased biomass production (Newcombe et al., 2001; Benetka et al., 2011). Poplar trees synthesize and accumulate high constitutive amounts of several classes of phenolic secondary metabolites such as salicinoids, flavan-3-ols [catechin and proanthocyanidins (PAs)], and phenolic acids and their esters in different tissues which are thought to defend against biotic and abiotic stresses (Palo, 1984; Donaldson et al., 2006; Tsai et al., 2006, Ullah et al., 2017). Salicinoids and PAs are usually the most abundant phenolic metabolites in poplar leaves (Lindroth and Hwang, 1996; Donaldson et al., 2006). The phytochemical make-up of stems, however, is less well known in poplar.

Flavan-3-ols, including monomeric catechins and oligomeric PAs (also known as condensed tannins), are major end products of the flavonoid biosynthetic pathway in many plant species (Dixon et al., 2005). Structurally, flavan-3-ols are very diverse due to the different hydroxylation patterns on the B ring, differing stereochemistry of the C ring 3-hydroxyl group, and different degrees of polymerization (Ferreira and Slade, 2002; Dixon et al., 2005). The biosynthesis of flavan-3-ols has been well studied in plants, including tree species (Bogs et al., 2005; Paolocci et al., 2007; Pang et al., 2013; Hammerbacher et al., 2014). The last steps of monomer biosynthesis are catalyzed by two distinct classes of enzymes. For the synthesis of 2,3-*trans*-flavan-3-ols (e.g., (+)-catechin), leucoanthocyanidins are reduced to form their corresponding flavan-3-ol (Tanner et al., 2003; Pang et al., 2013). This reaction is catalyzed by leucoanthocyanidin reductase (LAR). For the biosynthesis of the 2,3-*cis*-type flavan-3-ols (e.g., (−)-epicatechin), leucoanthocyanidins are converted to anthocyanidins by anthocyanidin synthase (ANS) and then reduced by anthocyanidin reductase (ANR) (Xie et al., 2004) to form the corresponding flavan-3-ol.

The biosynthesis of flavan-3-ols and PAs, as well as their regulation by transcription factors, are well studied in poplar leaves (Mellway et al., 2009; Yuan et al., 2012; Yoshida et al., 2015; Ullah et al., 2017). Although these metabolites accumulate constitutively in poplar, their biosynthesis is often induced by mechanical wounding and pathogen infections (Miranda et al., 2007; Mellway et al., 2009; Ullah et al., 2017). The role of PAs in defense against insect herbivores of poplar and other plant species is controversial and often ineffective (Hemming and Lindroth, 1995; Ayres et al., 1997; Boeckler et al., 2014). On the other hand, (+)-catechin and PAs are effective, inducible defenses against infection by leaf pathogens in poplar (Ullah et al., 2017; Wang et al., 2017). Poplar genotypes that accumulate constitutively high amounts of flavan-3-ol metabolites are better defended against infection by the biotrophic rust fungus *M. larici-populina* compared to genotypes accumulating low amounts of these compounds (Ullah et al., 2017). However, it is not clear whether a similar defense response exists in poplar stems or against fungal pathogens with different lifestyles. Recently we isolated a novel fungal species from a native black poplar species, *P. nigra* L, in northeastern Germany and named it *Plectosphaerella populi* (Crous et al., 2015). The fungus is very slow-growing, hemibiotrophic, and causes canker-like symptoms in black poplar branches upon successful infection. As defense responses in poplar stems have rarely been investigated, isolation of this pathogen provided an excellent opportunity to study the chemical defenses and hormonal regulation in this tissue upon fungal infection.

Plant hormones regulate complex signaling networks directing plant growth and development as well as defense responses against biotic and abiotic stresses (Santner et al., 2009; Pieterse et al., 2012). Typically, salicylic acid (SA) induces defense responses against biotrophic pathogens and piercing-sucking insect herbivores, while jasmonic acid (JA) activates plant defenses against necrotrophic pathogens and chewing insect herbivores (Thomma et al., 1998). In addition, hormonal crosstalk often fine-tunes the outcomes of plant defense responses against microbial pathogens. For example, the crosstalk between SA and JA is antagonistic in *Arabidopsis* (Vlot et al., 2009; Pieterse et al., 2012). Most research in this field has been conducted on herbaceous annuals, and it therefore remains unclear whether the same patterns are also applicable to perennial woody plant species. A recent study demonstrated that rust-infected polar leaves accumulate flavan-3-ols, SA, JA, and the JA-isoleucine conjugate (JA-Ile) in a complex time-dependent manner. Interestingly, SA was shown to activate flavan-3-ol biosynthesis in poplar leaves and reduced their susceptibility to rust infection, whereas JA did not have those effects (Ullah et al., 2019).

Among the many other plant hormones, cytokinins (CKs) have long been known to play important roles in plant growth and development including cell proliferation and differentiation (Sakakibara, 2006). More recent evidence suggests that CKs also modulate plant immunity in herbaceous plant species by interacting with other hormone signaling pathways (Choi et al., 2010; Großkinsky et al., 2011; Behr et al., 2012; O’Brien and Benkova, 2013; Schäfer et al., 2015). For example, CK activates the SA signaling pathway resulting in elevated plant defense responses against pathogens (Choi et al., 2010; Naseem et al., 2014; Jiang et al., 2013). CKs also modulate secondary metabolism in *Nicotiana* including eliciting an increased biosynthesis of antifungal compounds (Großkinsky et al., 2011; Brütting et al., 2017). Interestingly, plant pathogens also have the ability to synthesize CKs *de novo* that act as virulence factors resulting in increased host susceptibility (Pertry et al., 2009; Sardesai et al., 2013). In poplar (*Populus* × *canescens*), *ARR5* was shown to be a response regulator in the CK pathway and its activity decreased with leaf expansion as well as under drought stress conditions (Paul et al., 2018). Moreover, CKs were found to be responsible for cambial development as well as radial stem growth in poplar (*P. tremula* × *P. tremuloides*) (Nieminen et al., 2008). However, whether CKs also play a role in defense against pathogen infection in poplars and other woody plants remains unexplored.

Here we investigated how black poplar trees respond to *P. populi* infection, which causes a canker-like symptom on the stem. We focused our study on (+)-catechin and PA metabolites as they have been shown to play an important role in poplar defense against a foliar pathogen. We first investigated if the content of flavan-3-ols increased in poplar stems infected by *P. populi* and analyzed transcripts of biosynthesis genes in both infected and control trees over a time course of infection. Then we tested using *in vitro* assays whether the increased levels of flavan-3-ols are toxic to the fungus. To determine how the defense response and flavan-3-ol accumulation are regulated, the levels of various hormones were measured in infected and uninfected trees, including SA, JA and CKs. Finally, poplar trees were treated with CKs to determine if these substances play a role in modulating flavan-3-ol accumulation.

## Materials and Methods

### Plant Material and Growth Conditions

The black poplar genotype *Populus nigra* L. NP1 was used in the study (Ullah et al., 2017). Young poplar saplings were propagated from stem cuttings and grown in a greenhouse (20°C/18°C day/night temperature, 60% relative humidity, natural light conditions) in 2 L pots. The pots were filled with a standard substrate mix that contained 15.4% sand (0.7–1.2 mm), 3.8% lightweight expanded clay aggregate, and 80.8% Klasmann substrate with a pH of 5.0–6.0 (Klasmann-Deilmann, Geeste, Germany). Each 2 L substrate mix was fertilized with 2 g Triabon (Düngerexperte.de, Attenzell, Germany), 2 g Osmocote Exact Mini (Everris International B.V., Heerlen, The Netherlands), 0.3 g Mikromax (ICL GmbH, Nordhorn, Germany), and 0.3 g Radigen (Jost GmbH, Iserlohn, Germany). After the saplings had grown to 30–40 cm, they were fertilized every week with 0.1% Ferty^®^ 3 (Planta Düngemittel GmbH, Regenstauf, Germany). Two weeks before starting an experiment, the trees were transferred to a climate chamber (22°C/19°C day/night temperature, 60% relative humidity, 16 h/8 h light/dark cycle). Trees with a height of approximately 1 m were used in all experiments.

### Fungal Pathogen and Culture Maintenance

The fungus *Plectosphaerella populi* [C. Ullah, A. Hammerbacher, S.B. Unsicker & L. Lombard, sp. *nov.*] was originally isolated from naturally infected old-growth black poplar trees growing in a floodplain forest in northeastern Germany (Crous et al., 2015). The culture was maintained in Petri dishes using 3.9% potato dextrose agar (PDA) medium (Fluka-Sigma Aldrich, St. Louis, MO, USA) and grown at 22°C in darkness.

### Inoculation of Poplar Stems With *P. populi*

**Figure 1:**
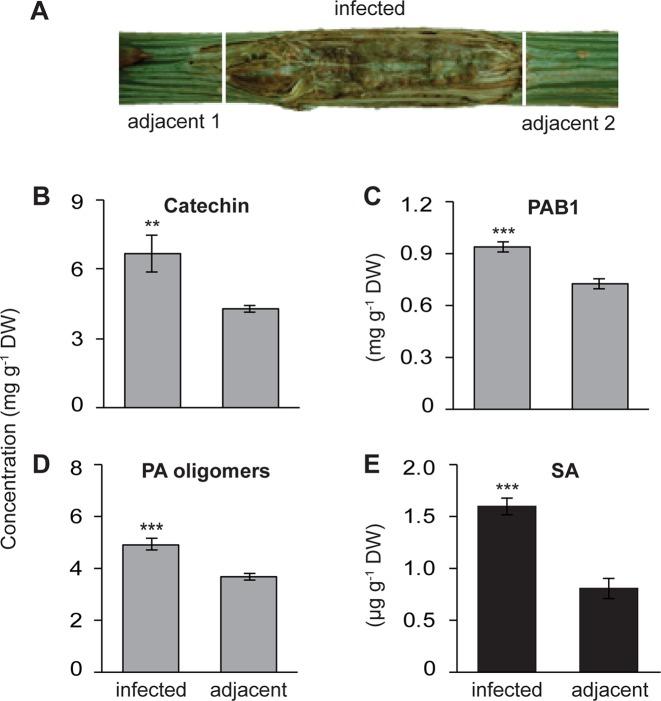
Increased local accumulation of catechin, proanthocyanidins (PAs) and salicylic acid (SA) in the stems of black poplar saplings after infection by *P. populi* compared to adjacent uninfected stem internodes. Poplar trees of approximately 1 m height were inoculated with the fungus. **(A)** Representative picture showing a *P. populi*-infected poplar stem with sampling areas. Infected stem internodes and adjacent uninfected internodes were harvested 6 weeks after inoculation. **(B**–**D)** Concentrations of the flavan-3-ol monomer (+)-catechin), the dimer PAB1, and oligomeric PAs. (+)-Catechin and PAB1 were analyzed by LC-tandem mass spectrometry. PA oligomers up to 10 monomeric units were measured by HPLC-FLD. The contents of PAB1 and PA oligomers were equivalent to the content of catechin. **(E)** Concentration of SA was analyzed by LC-tandem mass spectrometry. Data were analyzed using a paired t-test (**, *p* < 0.01; and ***, *p* < 0.001). Bars represent mean ± SE (n = 5). DW, dry weight.

Young *P. nigra* trees of approx. 1 m height and a stem diameter of 10–15 mm were chosen for inoculation with *P. populi*. Unless otherwise stated, each tree was inoculated at two positions: (i) internode between LPI 9-10 and (ii) internode between LPI 14-15. A small piece of bark (5 × 5 mm) was removed from the stem and a mycelial block (4 mm diameter) was placed into the wound. The control saplings were inoculated with only sterile PDA after removing a similar piece of bark to mimic the wound response. The inoculated portions were wrapped with Parafilm, which was then removed at 7 days post-inoculation (dpi). Samples were collected from separate trees at 10, 20, and 40 dpi. Two inoculated stem portions from the same plant were pooled and considered a single replicate. At each time point, five trees or replicates (n = 5) from both *P. populi* inoculated and wounded-control treatments were harvested. In a second experiment, both infected and adjacent stem internodes (**Figure 1A**) were collected from the same trees 6 weeks after fungal inoculation. Samples were immediately flash frozen in liquid nitrogen and stored in a −80°C freezer until further use.

### Extraction of Phenolics and Phytohormones From Poplar Stems

For extraction of flavan-3-ol metabolites as well as hormones, the stem internodes were ground with the help of a vibrating ball mill (Pulverisette 0, Fritsch GmbH, Idar-Oberstein, Germany) and lyophilized for 60 h at 0.22 mbar and −35°C. Approximately 50 mg lyophilized stem tissues were extracted with 1 ml analytical grade methanol, which contained 5 µg apigenin-7-glucoside (Carl Roth, Karlsruhe, Germany), and 4 µl phytohormone standard mix [40 ng of D_4_-SA (Santa Cruz Biotechnology, Santa Cruz, U.S.A.), 40 ng of D_6_-JA (HPC Standards GmbH, Cunnersdorf, Germany), and 8 ng D_6_-JA-Ile (HPC Standards GmbH)] as internal standards per ml. One ml extraction solvent was added to each micro centrifuge tube, vortexed vigorously, and incubated for 25 min at 20°C in a heating block shaking at 1000 rpm. The extracts were centrifuged at 11,000 ×g at 4°C for 5 min. Approximately 920 µl supernatant was transferred to a new micro centrifuge tube. The insoluble material was re-extracted with 1 ml 70% acetone. Both supernatants were combined and dried under a stream of nitrogen. The dried samples were re-dissolved in 0.5 ml methanol for 2 h at 4°C, centrifuged at 11,000 ×g at 4°C for 5 min. The supernatant was transferred to a new micro centrifuge tube and stored at −20°C.

### Quantification of Flavan-3-ol Monomers, PA Dimers, and PA Oligomers

Chromatography was performed on an Agilent 1200 HPLC system with the column eluent analyzed by a mass spectrometer. An API 3200 tandem mass spectrometer (Applied Biosystems, Darmstadt, Germany) equipped with a turbo spray ion source was operated in negative ionization mode. Separation was achieved on a Zorbax Eclipse XDB-C18 column (1.8 µm, 4.6 × 50 mm; Agilent). Formic acid (0.05%) in Milli-Q water and acetonitrile were employed as mobile phases A and B, respectively. The elution profile, mobile phase flow rate, column temperature, and all other parameters were similar to those described by Ullah et al. (2017). The parent ion and corresponding product ion of (+)-catechin, (−)-epicatechin, (+)-gallocatechin, PA dimers, and other flavonoids were analyzed by multiple reaction monitoring (MRM) as described by Ullah et al. (2017). Data acquisition and processing were performed using Analyst 1.5 software (Applied Biosystems). Flavan-3-ol concentrations were determined relative to the calibration curve established for apigenin-7-glucoside as an internal standard.

For oligomeric or polymeric PAs, the extract was diluted in methanol:acetonitrile (1:1) and was analyzed by an HPLC coupled to a fluorescence detector (FLD). PAs were separated on a LiChrosphere diol column (250 × 4 mm, 5 µm) (Merck Chemicals GmbH, Darmstadt, Germany) using an Agilent 1100 series HPLC employing a method previously described by Hammerbacher et al. (2014). PA oligomer and polymer concentrations were determined using a calibration curve for (+)-catechin and expressed as mg catechin equivalents.

### Reductive Cleavage of PAs

Reductive cleavage of PAs was carried out as previously described by Hammerbacher et al. (2014) and Ullah et al. (2017). Briefly, the same samples used for PA analysis were diluted 50-fold with methanol. The reaction was performed in HPLC glass vials containing 780 µl diluted extract, 20 µl trifluoroacetic acid, and 100 µl sodium cyanoborohydrate (0.5 g per ml methanol). Reaction mixtures were heated to 65°C for 15 min before adding additional 20 µl trifluoroacetic acid. Vials were sealed tightly and incubated overnight at 65°C. The following morning, the reaction was dried completely under a stream of nitrogen, re-suspended in 800 µl methanol, and centrifuged at 11,000 ×g for 5 min at 4°C. Approximately 780 µl supernatant was transferred to a new vial. The flavan-3-ol monomers, catechin, and epicatechin, were quantified by LC-tandem mass spectrometry as described above.

### Quantification of Phytohormones

For SA, JA, and JA-Ile quantification, chromatography was performed on an Agilent 1260 HPLC system (Agilent Technologies, Santa Clara, California, USA). An API 5000 tandem mass spectrometer (AB SCIEX, Darmstadt, Germany) equipped with a turbo spray ion source was operated in negative ionization mode. Hormones were separated on a Zorbax Eclipse XDB-C18 column (1.8 µm, 4.6 × 50 mm; Agilent) at 25°C, with two mobile phases consisting of 0.05% formic acid in water (solvent A) and acetonitrile (solvent B), at a flow rate of 1.1 ml min^–1^ following the elution profile described by Vadassery et al. (2012). The parent ion and corresponding product ions of SA and jasmonates were analyzed by MRM as described earlier (Vadassery et al., 2012). The concentrations of SA, JA, and JA-Ile were determined relative to the corresponding internal standard. The concentration of SAG was determined relative to the standard D_4_-SA applying a theoretical response factor of 1.0.

For CK and indole acetic acid (IAA) analysis, the same LC-MS/MS system was used. Hormones were separated on a Zorbax Eclipse XDB-C18 column (1.8 µm, 4.6 × 50 mm). Formic acid (0.05%) in water and acetonitrile were employed as mobile phases A and B, respectively. The elution profile was: 0–0.5 min, 5% B; 0.5–6.0 min, 37.4% B; 6.0–7.5 min, 100% B; 7.6– 10.0 min, 100% B. The flow rate of mobile phase was 1.1 ml min^−1^. An API 5000 tandem mass spectrometer was operated in positive ionization mode. The instrument parameters were optimized by infusion experiments with pure standards as previously described by Schäfer et al. (2014). The ion spray voltage was maintained at −5,500 V. The turbo gas temperature was set at 700°C. Nebulizing gas was set at 70 psi, curtain gas at 25 psi, heating gas at 60 psi, and collision gas at 6 psi. Multiple reaction monitoring was used to monitor analyte parent ion → product ion as follows: m/z 220.2 → 136.3 (collision energy [CE], 26 V; declustering potential [DP], −26 V) for zeatin; m/z 336.1 → 204.3 (CE, 61 V; DP, 61 V) for isopentenyladenine riboside (IPR) (for abbreviations see **Figure 6** legend); m/z 352.2 → 220.3 (CE, 76 V; DP, 76 V) for cZR; m/z 366.1 → 204.1 (CE, 116 V; DP, 116 V) for IP7G; m/z 382.1 → 220.2 (CE, 86 V; DP, 86 V) for tZ9G; m/z 514.1 → 220.3 (CE, 106 V; DP, 106 V) for cZROG; m/z 242.0 → 136.0 (CE, 50 V; DP, 50 V) for oT; m/z 176.0 → 130.0 (CE, 31 V; DP, 31 V) for IAA. Analyst 1.6 software (Applied Biosystems) was used for data acquisition and processing. The peak area of each hormone was normalized to the dry weight of the tissue and expressed as a relative level. To test if *P. populi* produces CKs, a 2-week-old actively growing fungal culture and a medium-only sample were homogenized separately in 2 ml safe-lock Eppendorf tubes containing 1 ml methanol using a micro pestle. Then supernatant was collected and analyzed following the methods described above.

### Histochemical Staining

*Plectosphaerella populi*-infected and adjacent stem internodes were embedded into Tissue Freezing Medium (Jung, Leica Biosystems, Wetzlar, Germany) and left for 2 h at −20°C. Sections (20 µm) were cut using a cryotome (CM1850, Leica Biosystems, Wetzlar, Germany), then stained for 8–10 min with 1% *p*-dimethylaminocinnamaldehyde (DMACA) solution, and observed under an inverted light microscope as described by Ullah et al. (2017).

### *In Vitro* Bioassays With *P. populi*

The culture medium for *P. populi* was prepared by dissolving potato dextrose agar powder (Fluka-Sigma Aldrich) in water following the manufacturer’s protocol. After autoclaving the medium, catechin or PAs (grape PAs, BIOTAN^®^, Laffort, Bordeaux Cedex, France) were added separately at a concentration of 2 mg ml^−1^ and heated to dissolve. Around 25 ml medium was dispensed in each Petri dish (diameter = 9 cm) and allowed to solidify. An agar plug (diameter = 4 mm) from a 2-week-old *P. populi* culture was placed in the middle of each Petri dish, sealed with Parafilm, and incubated at 22°C in a dark cabinet. The radial mycelial growth of the fungus was measured every 7 days until it reached the margins of the petri dish (n = 7).

### RNA Isolation, cDNA Synthesis, and Quantitative Real-Time PCR

Total RNA from finely ground stem tissue was extracted using the Invitrap Spin Plant RNA Mini Kit (Stratec Biomedical, Birkenfeld, Germany) following manufacturer’s instructions, except that an additional DNase treatment was included (RNase-Free DNase Set, Qiagen) as described by Ullah et al. (2017). Reverse transcription of 1 µg RNA into cDNA was performed by using SuperScript II reverse transcriptase (Invitrogen) and 50 pmol Oligo(dT)12-18 primer (Invitrogen) in a reaction volume of 20 µl. The cDNA was diluted up to a volume of 100 µl with sterile water. The Quantitative Reverse Transcription PCR (qRT-PCR) reactions were performed in a 20 µl volume as described by Ullah et al. (2017). Transcript abundance was normalized to the abundance of *Ubiquitin* (XM_006370571.2) and was calculated from five replicates (n = 5), with each replicate being analyzed from a mean of two technical replicates. Primer sequences used in this study are given in **Table S1**.

### Exogenous Spraying With Cytokinins

Zeatin (Sigma-Aldrich Chemie GmbH, Germany) was purchased to manipulate the endogenous CK levels in poplar trees. The product (CAS Number 13114-27-7) was dissolved in methanol to make a 5 mM stock solution. Then a 2.5 µM zeatin solution was generously sprayed onto the leaves and stem internodes of black poplar saplings until the liquid dripped off. The mock-treated plants were sprayed similarly but only with water containing 0.01% methanol. After 7 days, the stem internodes between LPI 5-10 were harvested, ground with the help of a vibrating mill in liquid N_2_. Flavonoids and phytohormones were extracted and analyzed as described above with the exception that only 10 mg freeze-dried tissue was used.

### Statistical Analysis

All data were analyzed by using the statistical package R version 3.2.0. Before analysis, normality and homogeneity of variances were tested using Shapiro-Wilk and Levene’s tests, respectively. Whenever necessary data were square root or log transformed to meet the assumptions for statistical testing. Flavan-3-ol and hormone metabolites in *P. populi*-infected and adjacent stem tissues of the same tree were analyzed by a paired t-test. Data obtained from *P. populi*-infected and wounded control trees at each time point, and CK vs. mock-treated trees, were analyzed using a two-tailed Student’s t-test.

## Results

### Levels of Catechin, PAs, and SA Increase in *P. populi*-Infected Tissues Compared to the Adjacent Stem Internodes

Poplar trees increase their synthesis and accumulation of the antifungal metabolites, catechin, and PAs, in leaves upon infection by an obligate biotrophic rust fungus (Ullah et al., 2017). To investigate whether a similar defense response is also activated in poplar stems against fungal infection, we used *P. populi*, a recently described fungal species with a hemibiotrophic lifestyle (Crous et al., 2015) that causes canker-like symptoms in poplar stems. Stem internodes (LPI 9-10) of five black poplar saplings were inoculated with *P. populi* and samples were collected 6 weeks after inoculation. Samples were harvested from the infected stem internodes and the adjacent stem internodes (**Figure 1A**). Finely ground stem tissues were extracted in methanol and 70% acetone, and analyzed by LC-MS/MS and HPLC-FLD.

The concentration of (+)-catechin, a 2,3-*trans*-flavan-3-ol with a 3’,4’-dihydroxylated B ring, increased significantly in *P. populi*-infected stems compared to the adjacent stem internodes (paired t-test, *p* < 0.01; **Figure 1B**). Similarly, the levels of PAB1 (catechin-epicatechin dimer) and PA oligomers were higher in the infected tissues than the adjacent tissues (Paired t-test, *p* < 0.001; **Figures 1C, 1D**). Flavan-3-ol monomers and PAs in plant tissues can be visualized by staining DMACA reagent, which produces a blue color (Abeynayake et al., 2011). Histochemical staining with DMACA revealed that monomeric flavan-3-ols and PAs accumulated significantly across the phloem and cambial zone of poplar stem internodes infected by *P. populi* in comparison with adjacent healthy stem internodes (**Figure S1**). Furthermore, the levels of the plant hormone SA increased approximately 2 times in fungus-infected tissues at 6 weeks post-inoculation compared to adjacent uninfected tissues (Paired t-test, *p* < 0.01; **Figure 1E**). These findings suggest that black poplar responds to this slow-growing fungus by accumulating higher amounts of flavan-3-ols and SA.

### Catechin and PAs Inhibit *P. populi* Growth *In Vitro*

**Figure 2:**
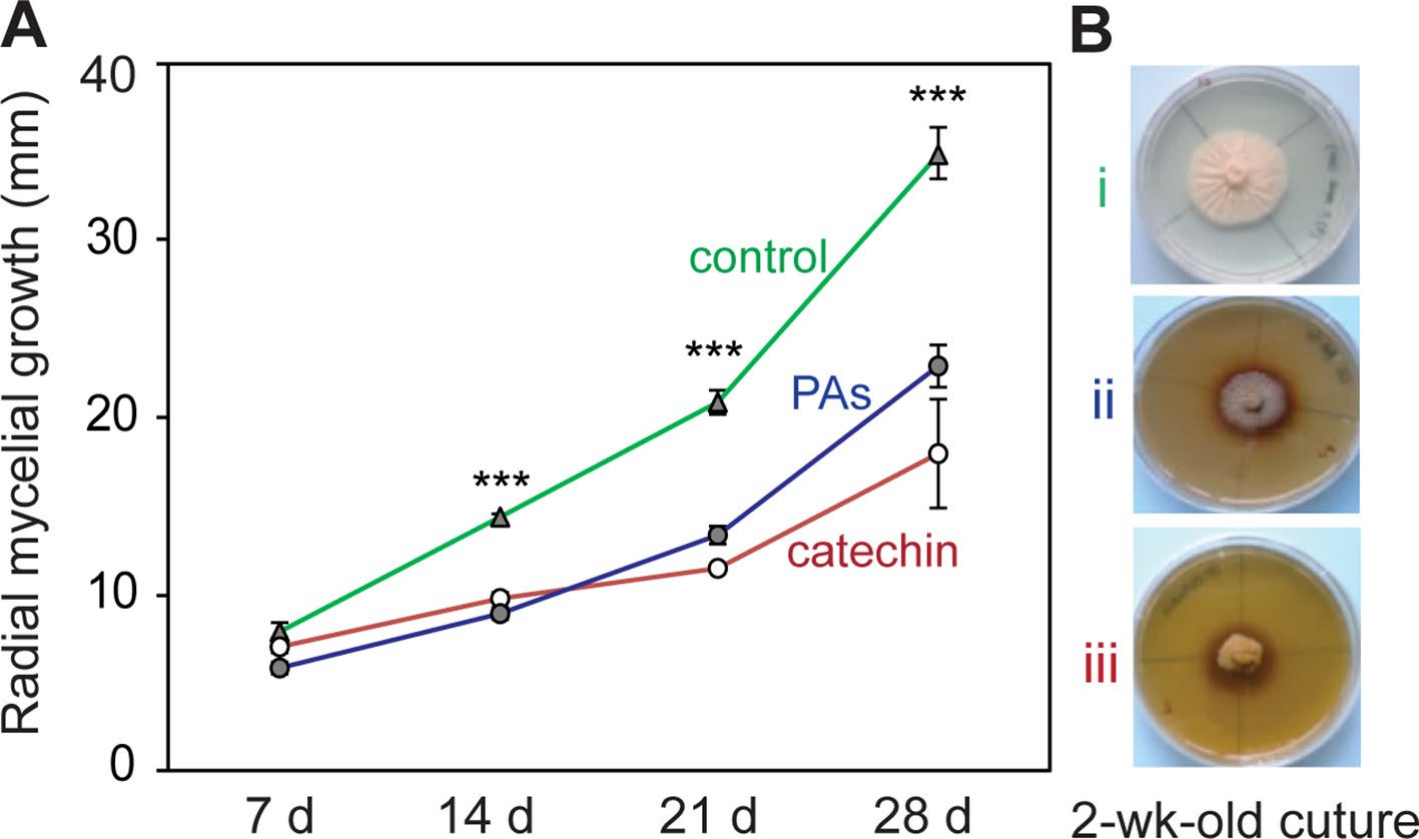
Catechin and proanthocyanidins (PAs) have antifungal activity against *P. populi*. **(A)** Radial mycelial growth of the fungus in culture medium supplemented with catechin or PAs at *in planta* concentrations. **(B)** Representative pictures of the fungus growing in culture media at 14 days: (i) control medium, (ii) PA medium (2 mg ml^-1^),and (iii) catechin medium (2 mg ml^-1^). Data were analyzed using Student’s t-test (***, *p* < 0.001). Data presented are mean ± SE (n = 7). d, day.

To determine whether the *in planta* concentrations of (+)-catechin and PAs have direct antifungal activities against *P. populi*, an *in vitro* bioassay was performed using these compounds. The fungus was grown on PDA medium supplemented with either catechin or PAs at the fresh weight concentrations found in infected tissues. A 4 mm diameter 2-week-old *P. populi* culture disk was inoculated in the center of the Petri dish and incubated in darkness. The radial mycelial growth of the fungus was slower on medium supplemented with catechin or with PAs compared to growth on the control medium (Student’s t-test, *p* < 0.001; **Figure 2**), indicating that these flavonoid metabolites are directly toxic to *P. populi*.

### Flavan-3-ol Monomers and PAs Accumulate in Black Poplar Stems Over a Time Course After *P. populi* Infection

**Figure 3:**
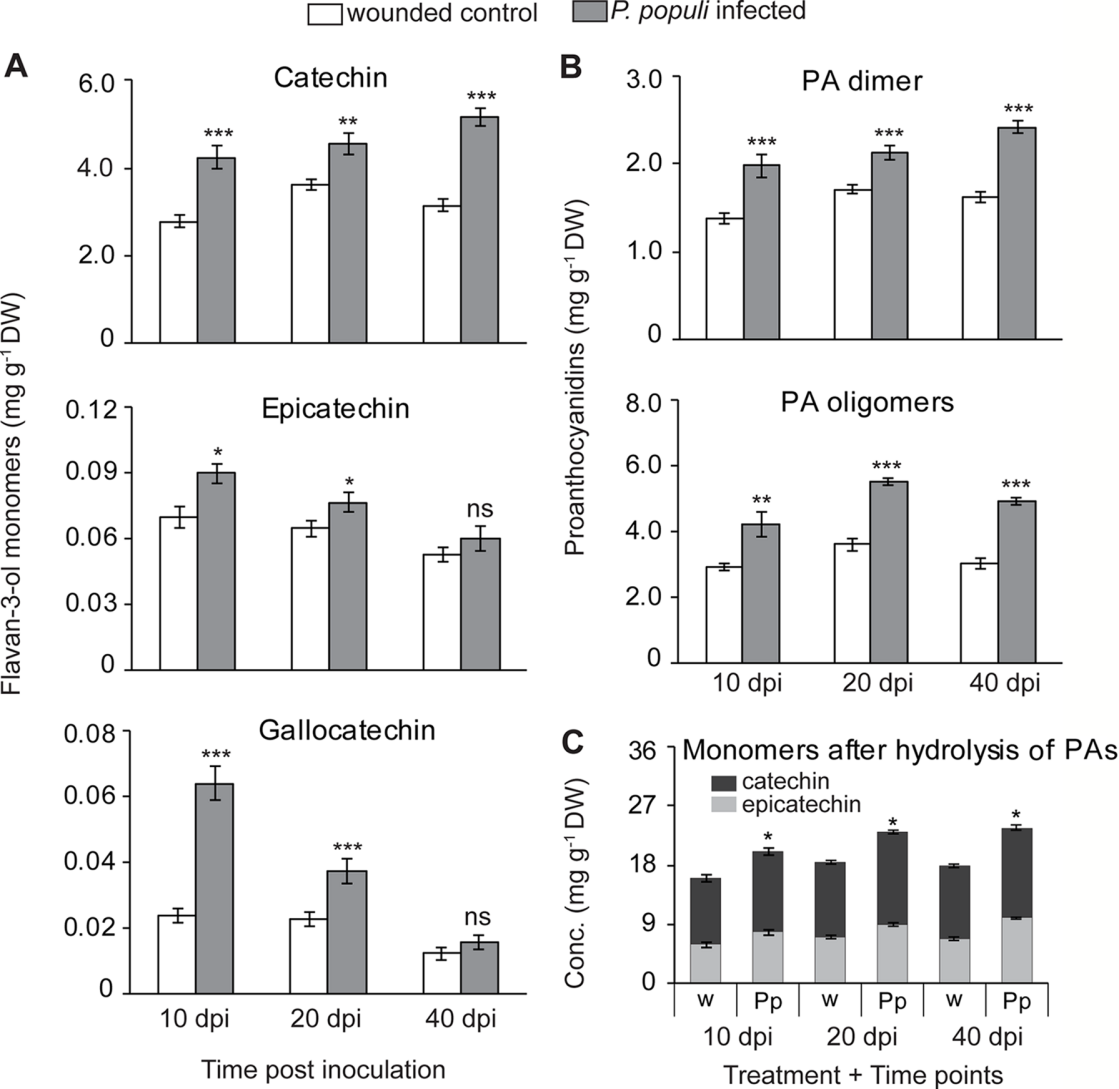
Flavan-3-ol monomers and proanthocyanidins (PAs) accumulate in black poplar stems in response to infection by *P. populi*. Concentrations of **(A)** flavan-3-ol monomers, **(B)** PA dimers and oligomers, and **(C)** flavan-3-ol monomers after hydrolysis of PA oligomers. Trees approximately 1 m in height were inoculated with *P. populi* after wounding and control trees were inoculated with the culture medium only. Samples were harvested from separate trees at each time point. Catechin, gallocatechin, and PA dimers were analyzed by LC-tandem mass spectrometry. PA oligomers were measured by HPLC-FLD. The levels of PAs were calculated as catechin equivalents. Data were analyzed using Student’s t-test (*, *p* ≤ 0.05; **, *p* < 0.01; and ***, *p* < 0.001; ns, non-significant). Bars represent mean ± SE (n = 5). dpi, days post inoculation, DW, dry weight; w, wounded; Pp, *P. populi*.

To expand our understanding of poplar responses to *P. populi* infection, we conducted a time-course infection experiment where wounded but non-infected controls were also included. Young black poplar trees were inoculated either with the fungus or with sterile culture medium after wounding. Samples were collected at 10, 20, and 40 days post-inoculation (dpi). The concentration of (+)-catechin increased significantly in poplar stems inoculated with *P. populi* throughout the infection period compared to the corresponding wounded controls (Student’s t-test, *p* < 0.01; **Figure 3A**). The highest accumulation was found in *P. populi*-infected stems at 40 dpi. The accumulation of another flavan-3-ol monomer, (−)-epicatechin, a 2,3-*cis*-flavan-3-ol with a 3’,4’-dihydroxylated B ring, was very low in poplar stems and only slightly increased after fungal infection 10 and 20 dpi (Student’s t-test, *p* < 0.05; **Figure 3A**). A 2,3-*trans*-flavan-3-ol monomer with a 3’,4’,5’-trihydroxylated B ring, (+)-gallocatechin, accumulated in significantly higher amounts at 10 and 20 dpi (Student’s t-test, *p* < 0.001) compared to the wounded control plants (**Figure 3A**).

PA dimers were predominantly procyanidin B1 (2,3-*cis*-(−)-epicatechin-(4β→8)—2,3-*trans*-(+)-catechin). These compounds accumulated to significantly higher levels in *P. populi*-infected stem tissues over the infection period compared to the wounded control trees (Student’s t-test, *p* < 0.001; **Figure 3B**). PA oligomers up to 10 monomeric units were also quantified by HPLC-FLD, and found to be significantly higher in *P. populi*-infected tissues compared to wounded control trees (Student’s t-test, *p* < 0.001; **Figure 3B**).

PAs differ in their composition of flavan-3-ol monomers, as well as the arrangement of starter and extension units (reviewed by Dixon et al., 2005). After reductive hydrolysis of poplar PAs into their respective monomeric units, high amounts of (−)-epicatechin were recovered but not (+)-catechin (**Figure 3C**). These results suggest that (−)-epicatechin is the major extender unit of PA chains in poplar tissues, whereas (+)-catechin is found mostly as a free monomer.

### Transcripts of Flavan-3-ol Biosynthetic Genes Increase in *P. populi*-Infected Stems

**Figure 4:**
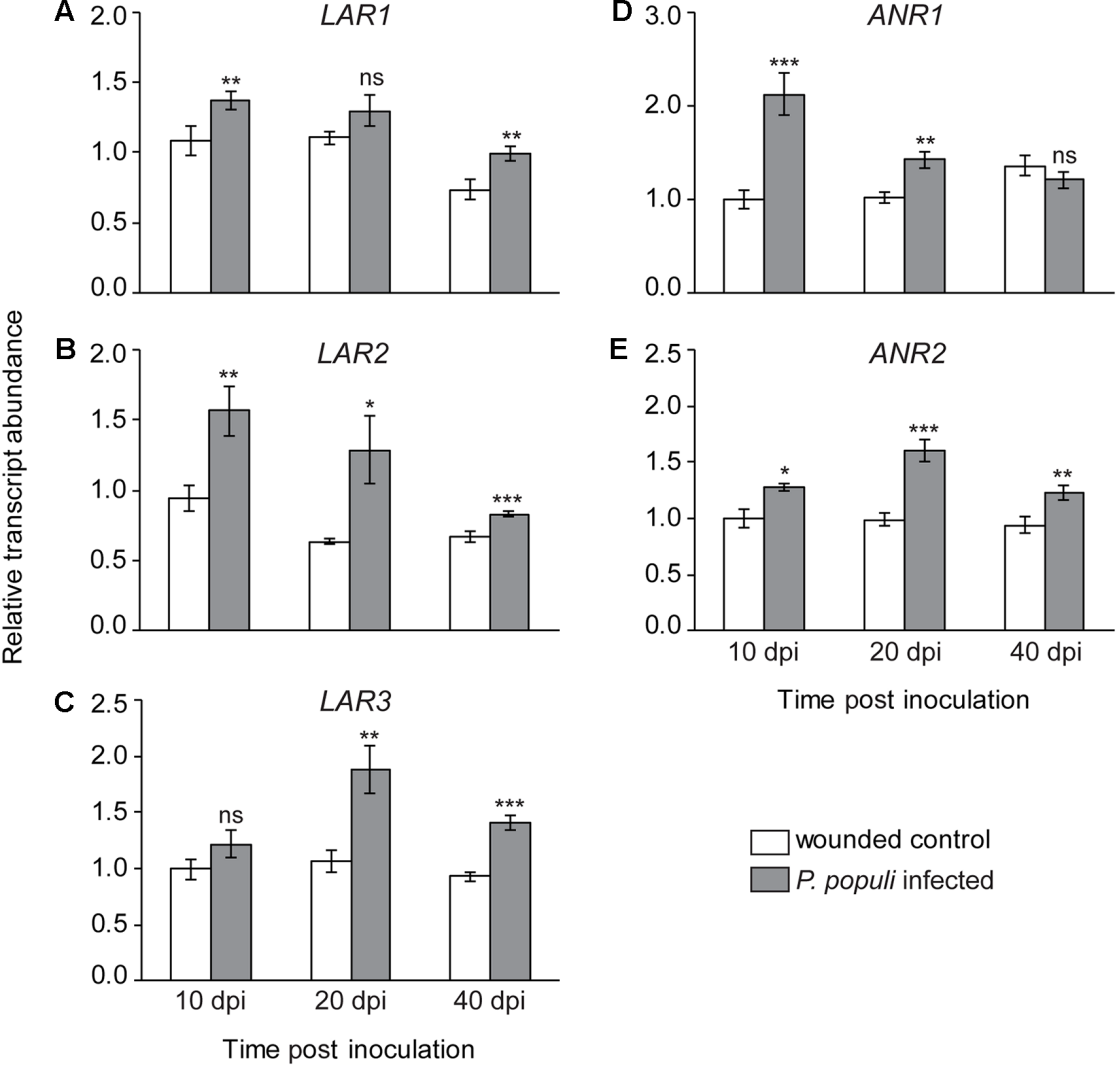
Transcript levels of flavan-3-ol biosynthesis genes. **(A**–**C)** Leucoanthocyanidin reductase (*LAR*) and **(D**–**E)** anthocyanidin reductase (*ANR*) transcripts increase in black poplar stems after infection by *P. populi* compared to wounded controls. The LAR and ANR enzymes are involved in catalyzing the last steps of 2,3-*trans*-(+)-flavan-3-ol (e.g., catechin) and 2,3-*cis*-(−)-flavan-3-ol (e.g., epicatechin) biosynthesis, respectively. The mRNA levels of *LAR* and *ANR* genes were measured by qRT-PCR using two technical replicates per sample. Transcript levels of each gene were normalized to *ubiquitin* expression. Data were analyzed using Student’s t-test (*, *p* ≤ 0.05; **, *p* < 0.01; and ***, *p < 0.001*; ns, non-significant). Bars represent mean ± SE (n = 5). dpi, days post inoculation.

To investigate the temporal dynamics of flavan-3-ol biosynthesis upon fungal infection in black poplar stems, we quantified the relative transcript abundance of *LAR* and *ANR* genes, which encode the enzymes that catalyze the last steps in flavan-3-ol monomer biosynthesis (Ullah et al., 2017). The relative transcript abundances of *LAR* genes, involved in the biosynthesis of *trans*-flavan-3-ols (e.g., catechin), were higher in *P. populi*-infected black poplar stems compared to the corresponding wounded control trees (**Figure 4**). The expression of the *LAR1* gene was significantly higher in the fungus-infected tissues at 10 and 40 dpi compared to the wounded controls (Student’s t-test, *p* < 0.01; **Figure 4A**), whereas *LAR2* transcripts were significantly higher over the whole course of infection compared to the wounded control saplings (Student’s t-test, *p* < 0.05; **Figure 4B**). On the other hand, the *LAR3* gene was significantly upregulated in *P. populi* inoculated stems at 20 and 40 dpi in comparison with the wounded controls (Student’s t-test, *p* < 0.01; **Figure 4C**). Transcripts of the *ANR* genes, which encode enzymes involved in the formation of *cis*-flavan-3-ols (e.g., epicatechin), were also higher in *P. populi-*infected poplar stems compared to the wounded control trees (**Figure 4**). While the transcript levels of the *ANR1* gene increased significantly in the infected trees at 10 and 20 dpi, the *ANR2* transcripts were significantly higher throughout the infection period in comparison with the corresponding wounded control trees (Student’s t-test, *p* < 0.05; **Figures 4D, E**). Our transcripts (**Figure 4**) and the corresponding metabolite data (**Figure 3**) indicate that black poplar increased flavan-3-ol biosynthesis and accumulated these defense metabolites in *P. populi*-infected stems throughout the infection period.

### *P. populi* Infection Induces SA, JA, and JA-Ile Accumulation in Black Poplar Stems

**Figure 5:**
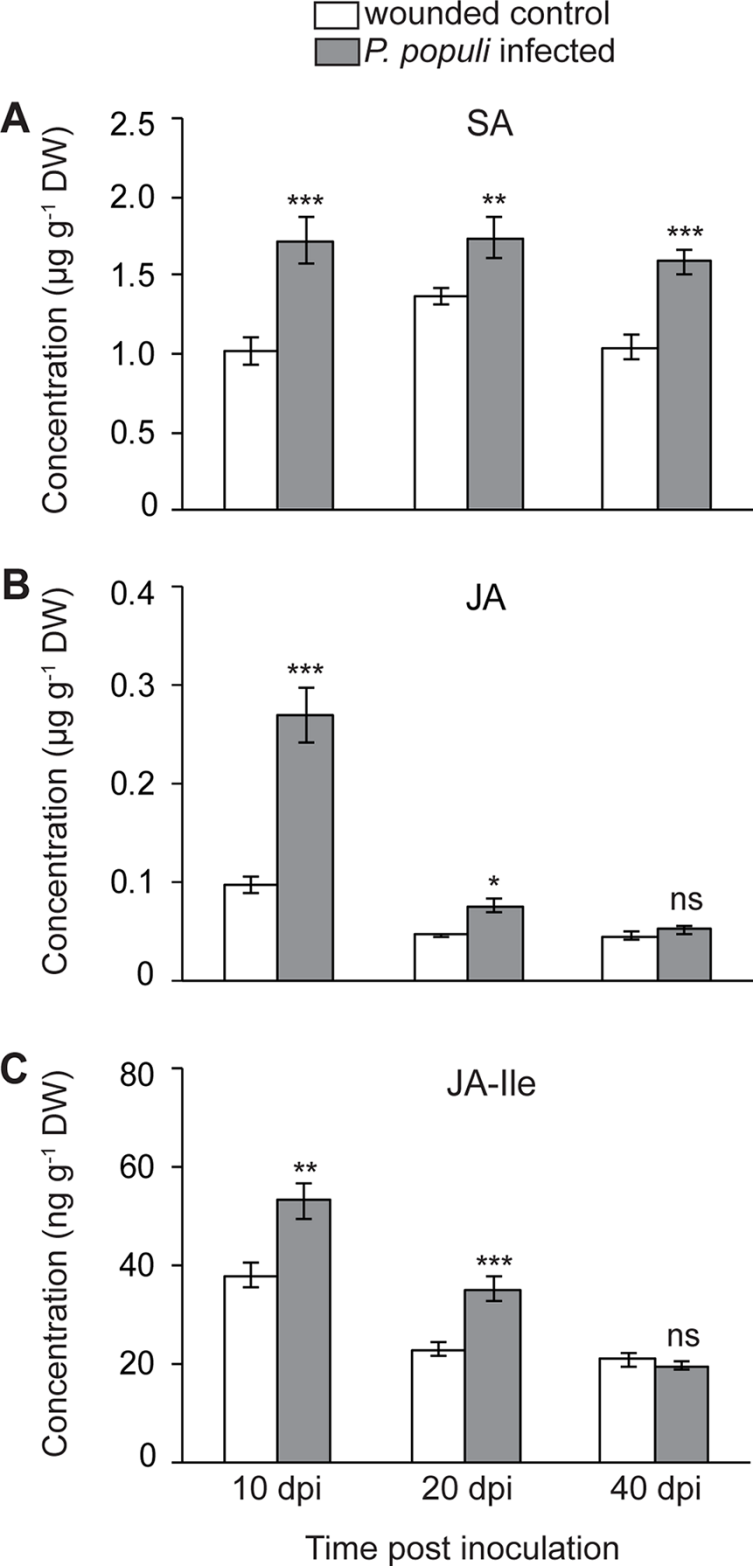
Concentrations of **(A)** salicylic acid (SA), **(B)** jasmonic acid (JA), and **(C)** JA-isoleucine (JA-Ile) in black poplar stems increase after *P. populi* infection. Trees approximately 1 m in height were inoculated with *P. populi* after wounding and control trees were inoculated with sterile culture medium. Analysis was performed by LC-tandem mass spectrometry. Samples were harvested from separate trees at each time point. Data were analyzed using Student’s t-test (*, *p* ≤ 0.05; **, *p* < 0.01; and ***, *p* < 0.001; ns, non-significant). Data represented in figures are mean ± SE (n = 5). dpi, days post inoculation; DW, dry weight.

It was shown that rust infection of poplar leaves leads to an upregulation of hormone signaling pathways in a time-dependent manner. SA activates flavan-3-ol accumulation resulting in reduced tree susceptibility to foliar rust infection, while JA did not appear to induce any defense responses to this pathogen (Ullah et al., 2019). In order to investigate whether *P. populi* infection also changes hormone levels, we analyzed SA and JA contents in stems infected by *P. populi* as well as in wounded controls. The levels of SA increased significantly in *P. populi*-infected stems over the course of the experiment in comparison with the wounded control trees (Student’s t-test, *p* < 0.01; **Figure 5A**). JA concentrations increased rapidly in *P. populi*-infected stem tissues compared to the wounded control trees (Student’s t-test, *p* < 0.001; **Figure 5B**), but the magnitude of its accumulation decreased during the later stages of infection at 20 dpi (*p* = 0.047) and 40 dpi (*p* = 0.92). Similarly, the levels of JA-Ile accumulated to significantly higher amounts in *P. populi*-infected stems compared to wounded control stems at 10 and 20 dpi (Student’s t-test, *p* < 0.01). However, this change was abolished at 40 dpi (Student’s t-test, *p* = 0.68; **Figure 5C**). We observed a sharp decline in JA and JA-Ile contents in both infected and wounded control trees over the course of the experimental period, whereas the SA content remained stable. To determine whether poplar stems in different growth stages accumulate different amounts of these hormones, we sampled stem internodes from three different positions (LPI 1-5, 6-10, and 21-22) from five control black poplar saplings. The concentration of SA did not change in stem tissues from different developmental stages. On the other hand, the contents of JA and JA-Ile were significantly higher in younger stem tissues and concentrations declined in older poplar stems (**Figure S2**).

### Cytokinins (CKs) Accumulate in Black Poplar Stems Infected by *P. populi*

**Figure 6:**
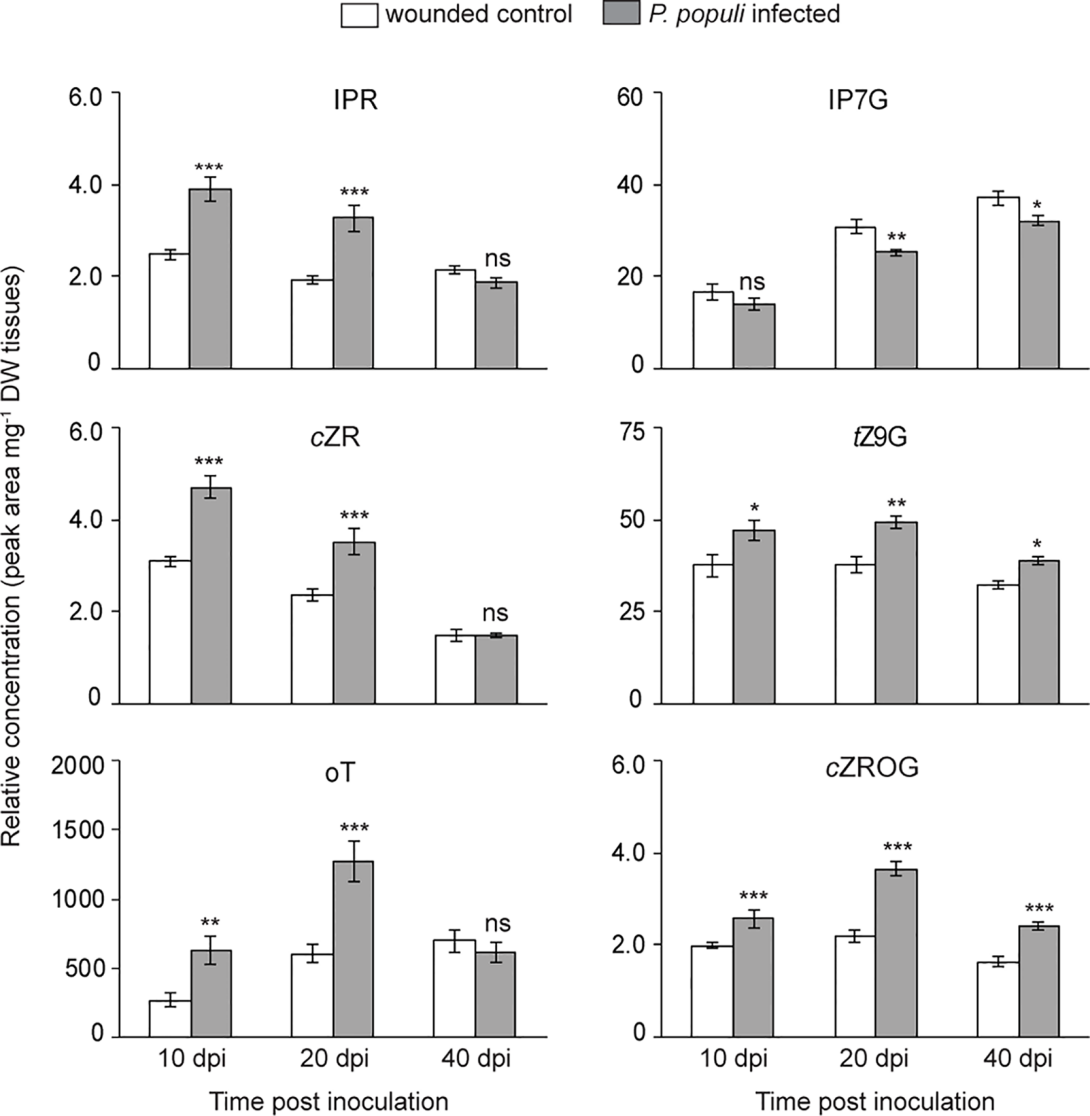
Cytokinins (CKs) accumulate in *P. populi*-infected poplar stems. Samples were collected from separate trees at each time point. Relative levels of CKs were quantified by LC-tandem mass spectrometry. Data were analyzed by Student’s t-test (*, *p* ≤ 0.05; **, *p* < 0.01; and ***, *p* < 0.001; ns, non-significant). Data presented in figures are mean ± SE (n = 5). IPR, isopentenyladenine riboside; cZR, *cis*-zeatin riboside; oT, *ortho*-topolin; IP7G, isopentenyladenine-N^7^-glucoside; tZ9G, *trans*-zeatin N^9^-glucoside; cZROG, *cis*-zeatin riboside-O-glucoside; dpi, days post inoculation; DW dry weight.

CKs and auxin play an important role in the regulation of plant growth and development as well as in stress responses (Sakakibara, 2006; Choi et al., 2011). Since *P. populi* infection in black poplar leads to an abnormal stem outgrowth characteristic of canker-like symptoms, we hypothesize that CKs and auxin could play an important role in this interaction. Therefore, we analyzed CK accumulation in both *P. populi*-infected and wounded control tissues. The levels of CKs such as isopentenyladenine riboside (IPR), *cis*-zeatin riboside (cZR), and *ortho*-topolin (oT) were significantly higher in *P. populi*-infected poplar stems in comparison with the wounded control stem internodes at 10 and 20 dpi (Student’s t-test, *p* < 0.001; **Figure 6**). Of the CK glucosides detected in poplar stems, *trans*-zeatin N9-glucoside (tZ9G) and *cis*-zeatin riboside-O-glucoside (cZROG), but not isopentenyladenine-N7-glucoside (IP7G) were present at significantly higher levels in *P. populi*-infected stem tissues compared to the corresponding wounded stem internodes over the course of infection (Student’s t-test, *p* < 0.05; **Figure 6**). On the other hand, the auxin IAA did not accumulate in *P. populi*-infected stems (**Figure S3**). It is well known that CKs are also produced by several microbes including some fungal pathogens as a virulence factor (reviewed by Spallek et al., 2018). To determine if *P. populi* produces CKs, we analyzed the fungal culture and compared this with sterile culture medium. However, we could not detect any plant CKs in the fungus or in inoculated culture medium. Therefore, our data suggests that CKs are indeed *de novo* synthesized by black poplar in response to *P. populi* infection.

### Exogenous CK Spraying Inhibits Flavan-3-ol Accumulation in Black Poplar

**Figure 7:**
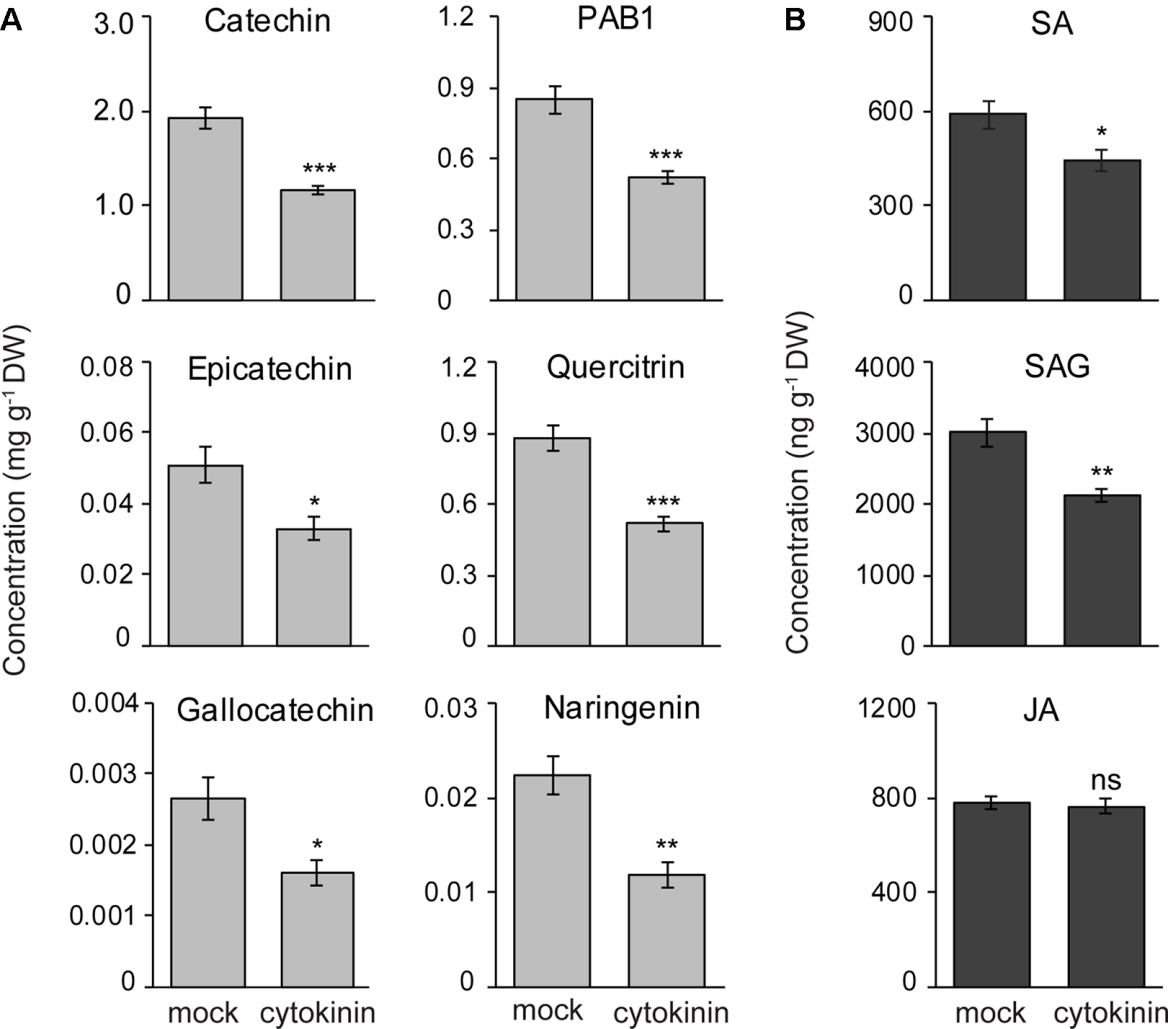
Exogenous cytokinin treatment of *P. nigra* resulted in lower accumulation of flavan-3-ol metabolites in stems. Poplar trees were treated with zeatin (2.5 µM). Stem samples were harvested from separate trees 7 days after spraying. The concentrations of **(A)** flavan-3-ols and some other flavonoids, and **(B)** phytohormones were monitored in poplar stems. Data were analyzed using Student’s t-test (*, *p* ≤ 0.05; **, *p* < 0.01; and ***, *p* < 0.001; ns, non-significant). Data presented in figures are mean ± SE (n = 8). PAB1, procyanidin B1; SA, salicylic acid, SAG, SA-glucoside; JA, jasmonic acid; DW, dry weight.

To determine if CKs regulate the accumulation of flavan-3-ol defense metabolites, we treated black poplar saplings with zeatin (2.5 µM) or with 0.01% methanol as a mock treatment. Stem internodes between LPI 5-10 were collected 7 days after spraying. The contents of flavan-3-ol monomers such as (+)-catechin (Student’s t-test, *p* < 0.001), (−)-epicatechin (Student’s t-test, *p* = 0.016), and (+)-gallocatechin (Student’s t-test, *p* = 0.012) all decreased in CK treated poplar stem internodes compared to the mock-treated control trees (**Figure 7A**). PAB1 also accumulated to significantly lower amounts in CK treated trees in comparison with mock-treated trees (Student’s t-test, *p* < 0.001; **Figure 7A**). We also quantified other flavonoids and found that quercitrin (*p* < 0.001) and naringenin (*p* < 0.01) contents decreased in CK treated trees (**Figure 7A**). These results suggest that CK negatively affects the accumulation of flavonoids in poplar.

To reveal if reduced flavan-3-ol accumulation in CK treated poplar saplings was due to changes in concentrations of other major defense hormones, we analyzed the contents of SA, SA-glucoside (SAG), and JA in poplar stems with or without CK treatment. Interestingly, the levels of SA (Student’s t-test, *p* = 0.028) and SAG (*p* = 0.002) decreased significantly in CK-treated stem internodes compared to mock-treated trees (**Figure 7B**). However, JA levels did not change due to CK treatment (Student’s t-test, *p* = 0.65) (**Figure 7B**). These results suggest that lower flavan-3-ol accumulation in black poplar stems treated with CK could be the result of a negative cross-talk between the SA and CK signaling pathways.

## Discussion

Although defense mechanisms in herbaceous plant species against pathogen infection have been investigated intensively in recent years, we know much less about the defense strategies of woody species. Poplar trees increase their biosynthesis and accumulation of PA polymers and their monomeric units, catechin and epicatechin, in leaves upon infection by pathogens (Mellway et al., 2009; Ullah et al., 2017; Wang et al., 2017). However, it is unclear how poplar trees defend against pathogens that infect stems. In this study, we investigated the response of young black poplar saplings (*Populus nigra* L.) to infection by *Plectosphaerella populi*, a recently described fungus that infects poplar stems (Crous et al., 2015). A strong accumulation of anti-microbial flavan-3-ols was observed in *P. populi*-infected stems compared to wounded, but uninfected control trees. To investigate the regulation of flavan-3-ol accumulation, we measured phytohormone levels and observed increases over the course of infection in SA, a hormone found to positively regulate flavan-3-ol accumulation in leaves infected with rust (Ullah et al., 2019). CK concentrations also increased, but downregulated flavan-3-ol and SA accumulation after exogenous application of the hormone to poplar stems.

### Flavan-3-ol Monomers, Dimers, and Oligomers Accumulate in Black Poplar Stems Upon Infection by *P. populi* as Antifungal Defenses

Poplar responds to a range of biotic and abiotic stresses by increased biosynthesis and accumulation of flavan-3-ols in a spatially localized and time-dependent manner. In this study, the contents of flavan-3-ol monomers and PAs increased locally in *P. nigra* stems upon infection with the fungus *P. populi* throughout the experimental period compared to the controls. In previous work, leaf infection by the rust fungus, *M. larici-populina*, also resulted in the accumulation of flavan-3-ols which reached a maximum level during the sporulation phase of the fungus (Ullah et al., 2017). Other studies have also shown that catechin and PAs accumulate in tree stems upon pathogen infection as anti-microbial defenses (Barry et al., 2002; Danielsson et al., 2011; Hammerbacher et al., 2019). In agreement, catechin and PAs accumulated densely around the point of inoculation in the bark and cambium tissues of *P. populi*-infected poplar stems compared to the adjacent stem internodes. Such a localized pattern of accumulation was also seen in Norway spruce infected with *Heterobasidion parviporum* (Danielsson et al., 2011). Infection by *P. populi* induces a characteristic stem outgrowth that looks like a canker. Staining and microscopy of this region clearly indicated that the flavan-3-ol metabolites accumulate in this outgrowth.

The steady accumulation of catechin and PA metabolites in *P. populi*-infected stem tissues over the course of infection prompted us to investigate whether the mRNA transcript levels of flavan-3-ol biosynthesis genes also increased steadily over this period. Interestingly, transcript levels of LAR and ANR genes, which are involved in flavan-3-ol biosynthesis, were greater in *P. populi*-infected tissues than in wounded controls at all measured time points throughout the infection period. In contrast, other studies showed that in response to foliar rust infection, poplars only transiently upregulate the transcription of several genes involved in flavonoid biosynthesis, reaching a maximum level at around 7 days post-inoculation (sporulation phase) after which their abundances sharply decreased (Miranda et al., 2007; Ullah et al., 2017).

To test whether the most abundant flavan-3-ol monomer, (+)-catechin, and PA oligomers have direct antifungal effects on *P. populi*, we supplemented fungal culture medium with these metabolites. Interestingly, both monomers as well as polymers were toxic to the fungus at physiologically relevant concentrations. In an earlier study, we showed that flavan-3-ols inhibited rust spore germination and hyphal growth *in vitro* (Ullah et al., 2017). Flavan-3-ols also inhibit growth and development of necrotrophic fungi, such as the appressorial melanization of *Colletotrichum kahawae*, causing coffee-berry disease (Chen et al., 2006) and *in vitro* growth of *Endoconidiophora polonica* and *Heterobasidion annosum* infecting conifers (Hammerbacher et al., 2014; Nemesio-Gorriz et al., 2016). The increased biosynthesis of flavan-3-ols during fungal infections as well as their direct toxicity to fungi therefore strongly support the hypothesis that flavan-3-ols are a general chemical defense in trees against fungal pathogens, regardless of their lifestyle.

### The Contents of SA and JA Increase Simultaneously in *P. populi* Inoculated Poplar Stems

Upon pathogen challenge, plants activate hormone signaling pathways, especially the SA and JA pathways, which regulate downstream defenses (Vlot et al., 2009; Pieterse et al., 2012). It is well established that the SA and JA signaling pathways are antagonistic to each other in the model plant, *A. thaliana* (Pieterse et al., 2012). However, simultaneous activation of SA and JA pathways has been reported in poplar leaves in response to insect herbivory (Clavijo Mccormick et al., 2014) as well as to rust infection (Ullah et al., 2019). In contrast to other plant species, SA signaling in poplar is independent of nonexpressor of pathogenesis-related genes 1 (NPR1) (Xue et al., 2013), the master regulator of SA-induced downstream defense responses in *A. thaliana* (Cao et al., 1997). Furthermore, SA has been shown to play a role in poplar secondary metabolism (Morse et al., 2007; Xue et al., 2013). For example, SA and its functional analog benzothiadiazole (BTH) have been shown to activate flavan-3-ol accumulation in poplar to protect trees against foliar rust infection (Ullah et al., 2019).

It was therefore of interest to investigate whether sustained catechin and PA accumulation are also coupled to SA signaling in *P. populi*-infected stems. Interestingly, we found a steady accumulation of SA over the course of infection. Thus, in agreement with our previous findings, SA most likely activated flavan-3-ol accumulation in black poplar stems. However, the contents of JA and JA-Ile also increased during the early stages of *P. populi* infection. Black poplar trees treated with methyl jasmonate (MeJA) showed only minor changes in SA and flavan-3-ol contents (Ullah et al., 2019). Furthermore, a recent study showed that *P. davidiana* detached leaves treated with MeJA showed significant increases in their SA content (Park et al., 2017). Therefore, fungal induction of JA signaling might lead to an increase in SA signaling with no clear antagonism between SA and JA in poplar.

### CKs Negatively Regulate Flavan-3-ol Accumulation in Poplar

CKs are one of the key hormones regulating plant growth and development (Sakakibara, 2006). In poplar stems, CKs are predominant in the cortical zone (Paul et al., 2016) and play an important role in cambial development and radial stem growth (Nieminen et al., 2008). Since the fungus *P. populi* causes a stem outgrowth in poplar, we hypothesized that CKs might accumulate during the course of infection. Interestingly, we found that both active and inactive CKs accumulated in *P. populi*-infected poplar stems compared to the wounded control trees. CKs are also known to play a role in plant-microbe interactions (Choi et al., 2011; Giron et al., 2013), such as in symbiotic nitrogen fixation (Gonzalez-Rizzo et al., 2006; Murray et al., 2007). In *Arabidopsis*, CKs increase resistance against *Pseudomonas syringae* infection *via* induction of the SA signaling pathway (Choi et al., 2010). High levels of cellular CKs in tobacco leaves resulted in simultaneous activation of the JA and SA pathways as well as an induction of a hypersensitive-like response (Novak et al., 2013). CKs in tobacco also increased resistance against the bacterial pathogen *P. syringae* through increased biosynthesis of phytoalexins, independent from SA signaling (Großkinsky et al., 2011). In a wild tobacco species, CKs regulated JA mediated defense signaling against herbivores and altered the levels of secondary metabolites (Schäfer et al., 2015; Brütting et al., 2017). All of these studies suggest significant roles of CKs in plant defense against biotic attack.

In contrast, certain plant pathogens also synthesize CKs and thus modulate the defense responses of their hosts. For example, *Agrobacterium tumefaciens*, which causes crown gall disease in many dicot plants, carries a CK biosynthesis gene (*ipt*) in its T-DNA resulting in high levels of CKs in host plants leading to rapid development of tumors (Chilton et al., 1977). *Plasmodiophora brassicae* synthesizes CKs, which are important for the development of clubroot symptoms (Devos et al., 2006; Siemens et al., 2006). Therefore, the increased CK levels in poplar stems infected by *P. populi* could be involved in tree susceptibility or resistance.

In this investigation, we applied a CK to determine its effect on the content of flavan-3-ols. The decreased accumulation of flavan-3-ols observed was accompanied by a decrease in SA, suggesting an increase in susceptibility. However, on *P. populi* infection there was a simultaneous increase in both CK and SA. This may arise from attempts by the fungus to manipulate host CK levels during the early stages of infection to promote canker development by downregulating flavan-3-ol biosynthesis. Nevertheless, the increased accumulation of SA and flavan-3-ols throughout the infection process suggests that SA regulation eventually overrides CK regulation. Further studies on the relation between fungal infection, CK signaling, and flavan-3-ol biosynthesis with finer spatial and temporal scales may help to resolve these inconsistencies.

## Conclusion

Monomeric flavan-3-ols and PAs accumulated in *P. nigra* stem internodes as a defense response to infection by *P. populi*, a recently described fungal pathogen causing cankers on poplar stems. These metabolites accumulated in the cortical regions of infected stems and strongly inhibited *P. populi* growth *in vitro*. Hence, flavan-3-ols, which are known to defend leaves against a biotrophic pathogen, also defend stems against a hemibiotrophic pathogen. Phytohormone analyses revealed a sustained increase in SA levels over the course of infection, whereas CK and JA contents increased at 10 and 20 dpi. While SA is known to be a positive regulator of flavan-3-ol accumulation, exogenous treatment with CKs reduced the accumulation of flavan-3-ols in poplar stems without altering the levels of other defense hormones, suggesting that CKs are a negative regulator of flavan-3-ol biosynthesis. Taken together, the steady accumulation of flavan-3-ols upon infection of *P. populi* implies that SA signaling elicits stronger responses in the tree compared to CK signaling during the interaction between black poplar and this pathogen.

## Data Availability Statement

All datasets for this study are included in the article/**Supplementary Material**.

## Author Contributions

CU, SU, JG, and AH conceived the study. CU and AH designed the experiments, CU performed all experiments, collected data, and analyzed the data. MR assisted in phytohormone analysis. CU wrote the manuscript, which was edited by JG and AH. All authors read, gave comments and approved the manuscript.

## Funding

This research was financially supported by the Max Planck Society (MPG) and Jena School for Microbial Communication (JSMC).

## Conflict of Interest

The authors declare that the research was conducted in the absence of any commercial or financial relationships that could be construed as a potential conflict of interest.
