# Supplementary material for: Accumulation of Catechin and Proanthocyanidins in Black Poplar Stems After Infection by Plectosphaerella populi: Hormonal Regulation, Biosynthesis and Antifungal Activity
Source: Front Plant Sci. 2019 Nov 15;10:1441. doi: 10.3389/fpls.2019.01441 (PMC6873352; doi:10.3389/fpls.2019.01441)
Supplement: Supplementary file 1 [file DataSheet_1.docx]

**Supporting Information**


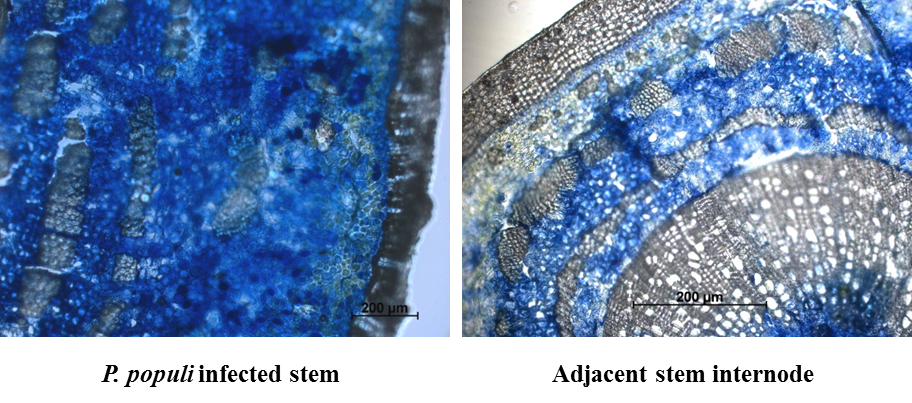


**Figure S1** Localization of flavan-3-ols and proanthocyanidins in black poplar stem internodes. Six-weeks after infection of black poplar stem internodes by *Plectosphaerella populi*, infected (left) and adjacent tissues (right) were used for making thin sections (20 µm) using a cryotome. The sections were stained with 4-dimethylaminocinnamaldehyde (DMACA) and observed under an inverted light microscope.


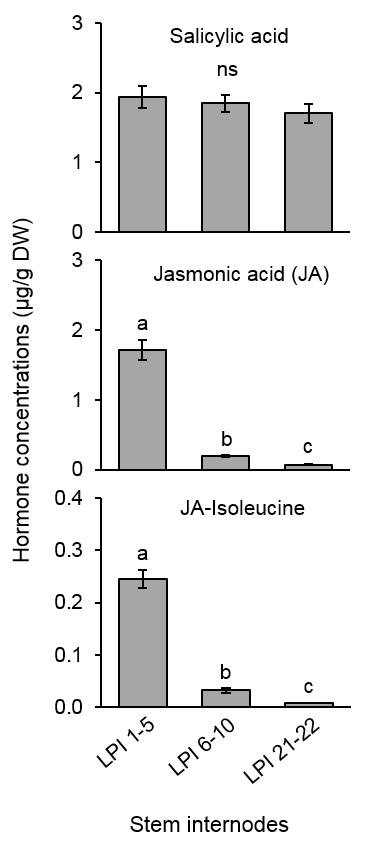


**Figure S2** Constitutive levels of salicylic acid and jasmonates in black poplar stems at different growth stages. Hormones were analyzed by liquid chromatography-tandem mass spectrometry (LC-MS/MS). Data were analyzed by one-way ANOVA followed by Tukey’s post-hoc test. Different letters indicate statistically different means (*p* < 0.05). Data presented in figures were mean ± SE (n = 5). LPI, leaf plastocron index; ns, non-significant; DW = dry weight.


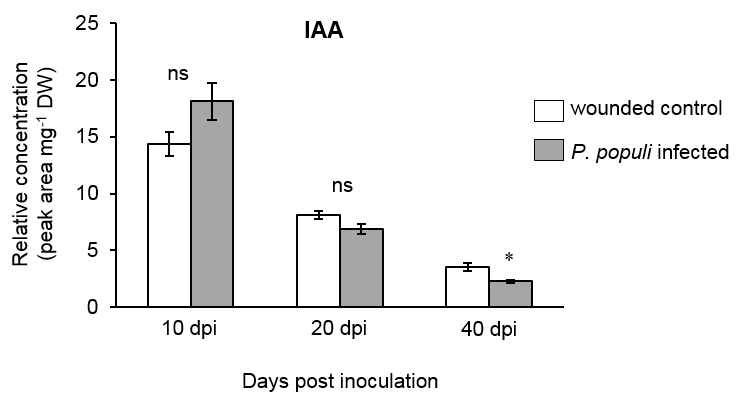


**Figure S3.** Relative amounts of auxin (indole acetic acid, IAA) in black poplar stems upon *Plectosphaerella populi* infection. Data were analyzed using Student’s t-test (*, *p* < 0.05; ns, non-significant). Bars represent mean ± SE (n = 5). DW, dry weight.

**Table S1** Primer sequences used in this study

| **Primer name** | **Primer sequence (5’ 🡪 3’)** |
| --- | --- |
| PnLAR1_qRT-for | CGAGTACTCATAGCCGGAGC |
| PnLAR1_qRT-rev | GGCTCCTTTGTCGTGAAGAG |
| PnLAR2_qRT-for | AACAAGTCGGTCCATTTTCG |
| PnLAR2_qRT-rev | GCA GCAATAGCAAGGAGGTC |
| PnLAR3_qRT-for | GAAGCTAGCCTCGAATGTGG |
| PnLAR3_qRT-rev | TTGGTCTGCTATGCTTGCAC |
| PnANR1_qRT-for | GCATCCCAGACCAAGAAAAA |
| PnANR1_qRT-rev | TCCCCCAAATTCTGTAGTGC |
| PnANR2_qRT-for | CCTGCCTCCAAGACACTAGC |
| PnANR2_qRT-rev | GCTGCTGGGAATATCTAGCG |
| PnUBQ_qRT-for | GTTGATTTTTGCTGGGAAGC |
| PnUBQ_qRT-rev | GATCTTGGCCTTCACGTTGT |
